# Patterns of systemic treatment for melanoma: An insight on trends and costs between 2019–2023 from the English systemic anti-cancer therapy national database

**DOI:** 10.1016/j.ejcskn.2024.100279

**Published:** 2025

**Authors:** Tommaso Bosetti, Oliver John Kennedy, Rebecca Lee, Avinash Gupta, Patricio Serra, Nadia Ali, Avanti Andhale, Sophia Kreft, Paul Lorigan

**Affiliations:** a The Christie NHS Foundation Trust, Manchester, UK; b Universitá degli Studi di Pavia, Pavia, Italy; c Division of Cancer Sciences, The University of Manchester, Manchester, UK

**Keywords:** Melanoma, Immune checkpoint inhibitors, Targeted therapy, Trends, Cost analysis

## Abstract

**Introduction:**

Checkpoint inhibitors (CPI) and targeted therapy (TT) have revolutionised the outcomes for melanoma. Other than the approval of pembrolizumab for resected stage IIB/IIC in February 2023, there were no changes in the Systemic Anti-Cancer Therapy (SACT) treatments available for melanoma in England between 2019 and 2023. The national SACT dataset provides an insight on systemic treatment use during this timeframe. The purpose of this study was to evaluate the patterns of use and costs of SACT for melanoma between 2019 and 2023.

**Materials and Methods:**

Data on prescriptions of SACT for adjuvant and metastatic disease between April 2019 to March 2023 were obtained from the SACT dataset and joinpoint regression analyses were used to look for any trends and change in trends. The list prices reported on the British National Formulary (BNF) were used to model drug acquisition costs.

**Results:**

Data were available from a total of 71 Hospital Trusts. There was a non significant increasing trend in the adjuvant prescriptions (semestral percentage change = 3.25, 95% confidence interval [CI] -2.15–8.96, p = 0.22) and a non significant negative trend in the metastatic prescriptions (semestral percentage change = -0.59, 95% CI -3.02–1.92, p = 0.64) from April 2019 to March 2023. The estimated costs for SACT in the same timeframe were approximately £ 1.2 billion. Despite an increase in the spending on adjuvant therapy, the total costs in the financial year 2022–2023 decreased compared to 2019–2020 due to a slight reduction in the spending on metastatic treatment.

**Conclusions:**

The opposite trends seen for adjuvant and metastatic prescriptions are a potential indicator of the impact of adjuvant treatment on development of distant metastases.

## 1 Introduction

Melanoma is the most aggressive form of skin cancer and the fifth most common cancer in the UK [1]. The incidence is rising in most European countries [2] and melanoma represents one of the most common cancers in young people [3]. Systemic treatment options have changed dramatically from 2011 onward with the advent of checkpoint inhibitors (CPI) and targeted therapy (TT) with BRAF/MEK inhibitors, revolutionising the outcomes for patients treated in both the metastatic and the adjuvant setting [4,5]. More recently, randomised clinical trials have shown that neoadjuvant treatment with CPI is superior to adjuvant treatment for clinically detectable stage III disease, and this is expected to become standard of care [6,7]. Furthermore, novel treatment options such as adoptive cell therapy with tumour infiltrating lymphocytes (TILs) have shown remarkable results and are already standard of care in the US and some European countries [8,9]. Innovative strategies such as mRNA-based individualised vaccine therapy have great promise and are currently being tested in phase 3 clinical trials.

Adjuvant treatment options available in England within the National Health Service (NHS) for American Joint Committee on Cancer Eighth Edition (AJCC-8) stage IIIA to IIID disease are single agent anti-PD-1 (pembrolizumab or nivolumab) and TT with dabrafenib plus trametinib in case of BRAF mutant disease. Adjuvant treatment is also available for patients with stage IIB/IIC (pembrolizumab) and radically treated stage IV (nivolumab). In the advanced setting, CPI can be given either as single agent (pembrolizumab, nivolumab or ipilimumab) or combination immunotherapy (ipilimumab plus nivolumab or nivolumab plus relatlimab). For BRAF mutant disease, TT with either dabrafenib plus trametinib or encorafenib plus binimetinib can be used. Chemotherapy has limited efficacy and is reserved for selected patients who have failed all the other lines of treatment [10].

Treatment with CPI and TT is associated with significant costs [11]. While a remarkable survival benefit for patients with advanced disease has been shown [12], the discussion around adjuvant treatment is more controversial [13,14]. Indeed, around 40–75% of patients eligible for adjuvant therapy would not experience distant recurrence with surgery alone [15], meaning that a significant number of patients undergo unnecessary systemic treatment. Also, despite the evidence of a reduction in the risk of developing locoregional or distant metastases, neither single agent anti-PD-1 nor TT have so far demonstrated a benefit versus placebo in overall survival. Financial sustainability of these treatments remains a topical issue.

In England, recommendations about systemic anticancer therapy (SACT) options are made by the National Institute for Health and Care Excellence (NICE) and the funding is provided by the NHS. The list price of the drugs is reported by the British National Formulary (BNF) and publicly accessible, but most of the drugs recommended by NICE are on the basis of a discount, which is commercial in confidence. The landscape of NHS available melanoma systemic treatment options in England has been relatively stable in the last five years. The approval of the new combination immunotherapy nivolumab plus relatlimab in 2024 and of pembrolizumab for patients with high-risk stage II disease in 2022 represent the first changes in years within the metastatic and adjuvant setting, respectively.

The SACT dataset available on the CancerStats2 platform is the world’s first comprehensive database collecting information on SACT on a national scale [16]. All NHS England Trusts providing SACT must submit monthly activity reports with information on the regimens prescribed, including the name of the regimen and the intent of treatment.

The purpose of this study was to evaluate the patterns of adjuvant and metastatic treatment for melanoma from 2019 to 2023 and to model the costs of these treatment to the NHS.

## 2 Materials and methods

Data on prescriptions of systemic treatment were obtained from the SACT dataset available on the CancerStats2 platform. As only public, aggregated data were considered, no ethics committee approval was required. The national prescription of SACT for melanoma from April 2019 to March 2023 was considered. This timeframe, based on the UK financial year, was selected because the dataset reports SACT activity from 2019 onward, and the information on the most recent months can be incomplete due to potential delays in data submission and processing time.

The number of new adjuvant and metastatic regimens in each semester (April to September and October to March) was collected. Semesters were chosen instead of years as at least seven data points were needed to test for changes in trends with the selected statistical analysis programme. A “new regimen” identifies the first prescription of a standard drug or combination of drugs and therefore reflects the start of a new line of treatment in either the adjuvant or metastatic setting in a selected time frame. In the metastatic setting, a new regimen can represent either the first or subsequent lines of treatment. Treatment intent is recorded on the dataset as “adjuvant”, “neoadjuvant”, “curative”, “palliative” or “other/unknown”. There was uncertainty about the term curative – even though this would mainly be attributed to adjuvant regimens, because of the improvements in treatment of advanced disease the intent to cure could also be applied to these regimens depending on tumour characteristics. To limit the confounding impact from the curative regimens and those with other/unknown or missing intent of treatment, only NHS Trusts where “adjuvant” and “palliative” regimens accounted for more than 80% of the total regimens were included in the trend analysis. In our analysis, the term “metastatic” was applied to the “palliative” regimens given the potentially curative intent of treatment for a significant subset of patients with advanced disease.

The administration count of the different drugs per financial year (April to March) was also collected. The term “administration” identifies the physical administration of the single drug(s) of a regimen, which reflects the number of cycles under that regimen given in a selected time frame. The administration count was then combined with the list price of the different drugs as reported on the BNF to model the costs of treatment for the NHS. It is important to note that normally these drugs are further discounted in the NHS, however information regarding actual price is not available. For cost modelling, when the dose of the drug was based on the patient’s weight (as with ipilimumab and with nivolumab when given in combination with ipilimumab), 75 kg was chosen as standardized weight. For those drugs given as a flat dose but available in different doses according to the selected schedule (pembrolizumab and nivolumab), the schedule commonly adopted in the clinical practice was arbitrarily chosen (pembrolizumab 400 mg Q6W and nivolumab 480 mg Q4W).

Joinpoint regression analyses were used to test for trend changes and compute the semestral percentage change in the prescription of new adjuvant and palliative regimens (Joinpoint Regression Program, Version 5.2.0, June 2024; Statistical Methodology and Applications Branch, Surveillance Research Program, National Cancer Institute). The joinpoint program uses an algorithm to test whether a multi-segmented line is a significantly better fit then a straight or less-segmented line to represent data. Line segments are joined at points called joinpoints. When identified, a joinpoint marks a trend change, and a period between two joinpoints represents a trend. The statistically best fitting model (0 versus 1 joinpoints) are presented. P values < 0.05 were considered statistically significant.

## 3 Results

A total of 71 NHS Hospital Trusts in England involved in SACT prescription for melanoma from April 2019 to March 2023 were identified. All the NHS Trusts reported consistently their SACT activity during the whole time period considered. The breakdown by intent of treatment for all NHS Trusts showed that metastatic and adjuvant regimens accounted for 90% of the treatments overall (67% and 23%, respectively). The intent of treatment of the remainder regimens was either curative (5%), neoadjuvant (1%), or other/unknown (4%), and was missing in 1%.

When considering the number of new treatments, nine NHS Trusts (13%) were excluded from this part of the study because the regimens with curative, neoadjuvant, other/unknown and missing intent of treatment accounted for more than 20% of the total regimens. The prescriptions from the excluded NHS Trusts accounted for 13% of all the total prescriptions.

**Fig. 1:**
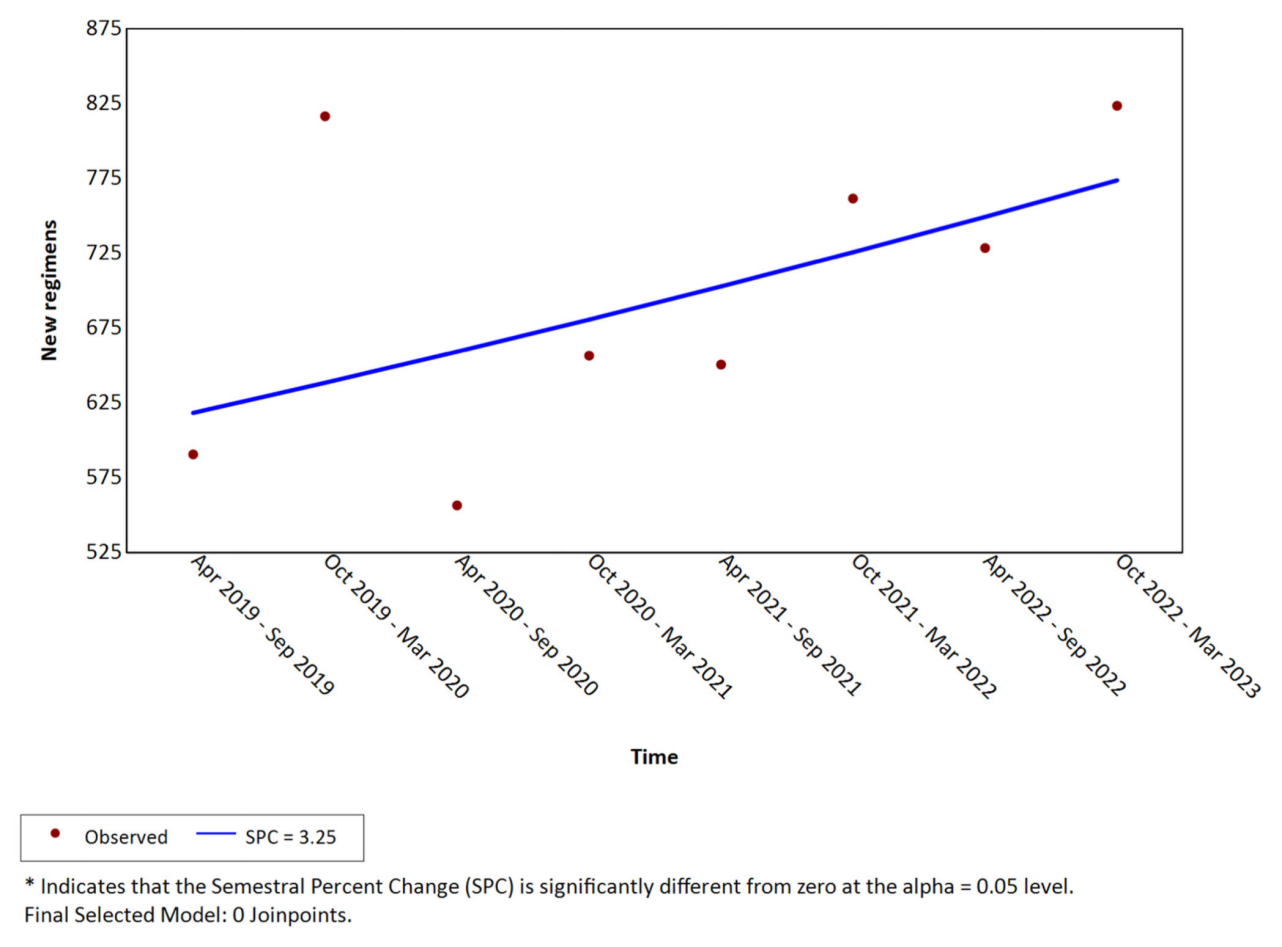
New adjuvant regimens count per semester.

The number of new adjuvant regimens per semester from April 2019 – September 2019 to October 2022 – March 2023 within the selected 62 NHS Trusts are shown in Fig. 1. There was a non statistically significant trend towards increased number of prescriptions over the entire time period studied (semestral percentage change = 3.25, 95% confidence interval [CI] -2.15–8.96, p = 0.22).

The breakdown for adjuvant regimens is shown in Table 1. CPI (nivolumab or pembrolizumab) and TT (dabrafenib plus trametinib) accounted for 72% and 28% of the new adjuvant regimens prescribed from April 2019 to March 2023, respectively, and this proportion was relatively consistent over the study period.

**Fig. 2:**
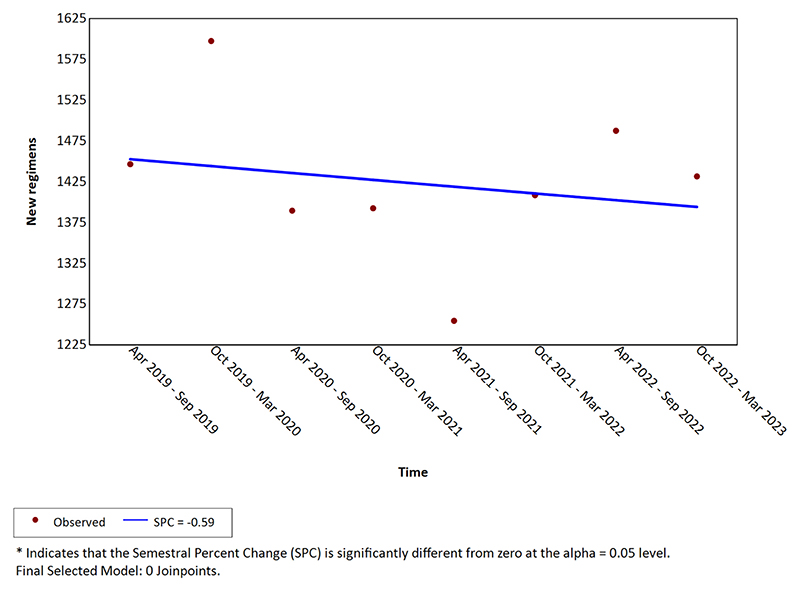
New palliative regimens count per semester.

The number of new metastatic regimens per semester in the same timeframe (including first and subsequent lines of therapy) is shown in Fig. 2. There was a slight and non significant trend towards decreased number of prescriptions over the entire time period studied (semestral percentage change= -0.59, 95% CI -3.02–1.92, p =0.64).

The breakdown for the main metastatic regimens is shown in Table 2. The prescription of ipilimumab plus nivolumab increased from 2020–2021 onwards, as opposed to single agent pembrolizumab. Single agent nivolumab is not reported in the breakdown because the count of nivolumab included both the regimens where nivolumab was given as single agent upfront and those where it was given as maintenance therapy after the induction phase with ipilimumab. However, similarly to the patterns of adjuvant treatment, in the metastatic setting nivolumab is usually only used as maintenance therapy because of the more frequent administration schedule compared to single agent pembrolizumab.

The number of cycles administered and the cost modelling based on the drug acquisition costs are shown in Table 3, Table 4 for adjuvant and metastatic treatment, respectively. The cost for combined adjuvant and metastatic treatment from April 2019 to March 2023 was approximately £ 1.2 billion. Palliative and metastatic treatment accounted for 68% and 32% of the costs, respectively. Despite a rise in the amount spent on adjuvant treatment, total costs in 2022–2023 decreased compared to 2019–2020 due to a reduction in the amount spent on metastatic treatment (Table 5).

## 4 Discussion

Our study provides an insight into SACT prescribing for melanoma in a timeframe where the landscape of treatment options was relatively stable. Within the time frame analysed, all the current regimens were already NICE approved before the start of our data collection (April 2019), except for pembrolizumab for high-risk stage II disease.

The drop in new SACT prescriptions in the semester April 2020 – September 2020 reflects the impact of the COVID-19 outbreak. This reduction was greater for adjuvant than for metastatic treatment (-31% and -13% vs. the previous semester, respectively), reflecting the guidelines issued on the use of SACT at this time [17]. The reason for the increase in both the adjuvant and metastatic prescriptions in October 2019 – March 2020 compared to the previous semester is unclear, as no new regimens were approved or clinical guidelines published at this time.

The absence of a change in the trends of adjuvant and metastatic treatment indicates that the pattern of SACT prescription has been relatively stable. However, the opposite trends in the adjuvant and advanced setting, although only mild and not statistically significant, raise interesting considerations. The positive trend in the adjuvant setting suggests that the number of patients started on adjuvant treatment has been rising, in keeping with the increasing incidence of melanoma in England [18] and the delayed diagnoses during the pandemic [19] leading to resection of tumours at a later stage. In this regard, there was little impact from the approval of adjuvant therapy for resected stage IIB/IIC disease as this only became available in February 2022 and initial uptake was low. On the other hand, the observed slight negative trend in the metastatic prescriptions might be an early indicator of the reduced risk of developing distant metastases following adjuvant treatment [20–23], even though this will need more time to be confirmed. In addition, the observed increasing use of ipilimumab plus nivolumab may also have resulted in fewer subsequent lines of treatment, due to the higher and prolonged disease control rate when compared to single agent anti-PD-1 and TT and the paucity of other treatment options when ipilimumab is used in the first line, especially for BRAF wild type patients.

No information on patient level data, including stage and BRAF mutational status, was available. However, assuming a 40% prevalence of the BRAF mutation [24], the observed relative contribution of adjuvant treatment with CPI and TT (72% vs. 28%) suggests that most patients with BRAF mutant disease were treated with TT. As no direct trial comparison exists and the efficacy between the two options in this setting is similar [25], the preference for TT can be explained by the different profile of side effects (particularly chronic endocrine toxicity) and the potential for CPI to be more effective in providing a durable response in the advanced disease setting.

The breakdown per regimen shows that pembrolizumab was the most prescribed drug in the adjuvant setting, driven largely by the 3-weekly or 6-weekly schedule as opposed to the 2-weekly or 4-weekly schedule for nivolumab. In the metastatic setting, combination immunotherapy ipilimumab plus nivolumab has been increasingly prescribed from 2020–2021 to 2022–2023, in contrast to TT. This pattern may reflect the impact of data from the DreamSeq and SECOMBIT trials, which were first presented in 2021 [26,27].

It is noteworthy that despite the rise in the costs of adjuvant treatment, the amount spent on SACT decreased from 2019–2020 to 2022–2023 thanks to a reduction in the spending on treatment for advanced disease. To date, in contrast to metastatic treatment, adjuvant treatment has a fixed duration of one year, and when immunotherapy is given, single agent anti-PD-1 is used. Preventing a recurrence can save multiple lines of longer and more expensive future palliative treatment [28]. This needs consideration even more so now that novel options such as TILs have become available, with treatment costs expected to exceed $500,000 [29]. Nevertheless, the discussion on adjuvant treatment cannot overlook the evidence that many patients are already cured by surgery alone, and in some cases the absolute benefit in the reduction of distant metastases remains low (around 10–15% according to the stage) [20–23]. In addition, none of the adjuvant pivotal trials has reported a survival benefit so far. Therefore, the expected benefit from preventive treatment will always need to be balanced against the risk of recurrence and toxicity, and identification of biomarkers to aid the selection of patients remains an unmet need. Lastly, in addition to the fewer new metastatic prescriptions, both discontinuation after serious adverse events and the growing tendency to stop the treatment after two years for patients with deep response [30] could be contributing to the reduction in the spending on metastatic treatment. Interestingly, both scenarios are more likely associated with the increasingly prescribed ipilimumab plus nivolumab combination.

This study has several limitations. First, there is some concern about data quality and completeness, related to data reporting and processing. The impact on the trend analysis from the exclusion of the regimens labelled as curative and with other/unknown intent of treatment was little as their number was low (10% of the regimens). On the other hand, the inclusion of both single agent and maintenance nivolumab under the count of nivolumab implies that the number of new metastatic regimens was slightly overestimated. Finally, no information about schedule and dose of treatment was provided on the dataset. This had no implication on the trend analysis. For costs modelling, though, the choice of considering 480mg and 400mg for nivolumab (single agent) and pembrolizumab, respectively, could have led to an overestimate of the spendings for SACT, assuming that nivolumab 240mg and pembrolizumab 200mg were also prescribed. This and the above-mentioned lack of public data on the actual acquisition price for the NHS make the costs presented most likely overestimated.

## 5 Conclusions

The suggestion in this study of opposite trends for adjuvant and metastatic treatment and of decreasing costs for SACT are intriguing, as this could be an indicator of the benefit from adjuvant therapy. Other potential explanations such as new patterns in the prescriptions for advanced disease (regimen choice and duration of treatment) need to be considered. It will be important to continue to monitor these trends and costs in the future to evaluate the impact of treatment for resected stage II disease. Identification of biomarkers for better selection of patients and consideration of de-escalation of adjuvant treatment could improve further the expenses on SACT.

## Figures and Tables

**Table 1:** New adjuvant regimens per financial year.

Year	Pembro n (%)	Nivo n (%)	Dab+Tram n (%)
*2019–2020*	716 (51)	234 (17)	373 (26)
*2020–2021*	630 (52)	165 (14)	356 (29)
*2021–2022*	764 (54)	188 (13)	417 (30)
*2022–2023*	849 (55)	229 (15)	413 (27)

Pembro = pembrolizumab; Nivo = nivolumab; Dab+Tram = dabrafenib plus trametinib.

**Table 2:** Selected new metastatic regimens per financial year.

Year	Ipi+Nivo n (%)	Pembro n (%)	Ipi n (%)	Dab+Tram n (%)	Enco+Bini n (%)
*2019–2020*	566 (19)	835 (27)	147 (5)	420 (14)	150 (5)
*2020–2021*	497 (18)	761 (27)	167 (6)	328 (12)	270 (10)
*2021–2022*	629 (24)	674 (25)	124 (5)	325 (12)	214 (8)
*2022–2023*	861 (29)	613 (21)	134 (5)	280 (10)	189 (6)

Ipi+Nivo = ipilimumab plus nivolumab; Pembro = pembrolizumab; Ipi = ipilimumab; Dab/Tram = dabrafenib plus trametinib; Enco/Bini = encorafenib plus binimetinib.

**Table 3:** Adjuvant drug administration count and costs per financial year.

	*2019–2020*		*2020–2021*		*2021–2022*		*2022–2023*	
	Count	Cost (£)	Count	Cost (£)	Count	Cost (£)	Count	Cost (£)
**Nivo**	3206	16,881,514	1345	7,082,232	1435	7,556,136	1869	9,841,406
**Pembro**	4605	48,444,600	4157	43,731,640	5000	52,600,000	5675	59,701,000
**Dab**	2855	15,988,000	2987	16,727,200	2928	16,396,800	2977	16,671,200
**Tram**	2784	12,472,320	2929	13,121,920	2895	12,969,600	2901	12,996,480
**Total**	13450	93,786,434	11418	80,662,992	12258	89,522,536	13422	99,210,086

Nivo = nivolumab; Pembro = pembrolizumab; Dab = dabrafenib; Tram = trametinib.

**Table 4:** Metastatic drug administration count and costs per financial year.

	*2019–2020*		*2020–2021*		*2021–2022*		*2022–2023*	
	Count	Cost (£)	Count	Cost (£)	Count	Cost (£)	Count	Cost (£)
**Ipi (combination with Nivo)**	1464	24,705,000	1248	21,060,000	1625	27,421,875	2111	35,623,125
**Nivo (combination with Ipi)**	1687	1,387,979	1419	1,167,482	1869	1,537,719	2527	2,079,089
**Nivo (single agent)**	7644	40,250,246	6527	34,368,571	6200	32,646,720	6069	31,956,926
**Pembro**	8123	85,453,960	5302	55,777,040	5900	62,068,000	5601	58,922,520
**Ipi (single agent)**	608	10,260,000	675	11,390,625	566	9,551,250	364	6,142,500
**Dab**	4642	25,995,200	3834	21,470,400	4288	24,012,800	4173	23,368,800
**Tram**	4516	20,231,680	3762	16,853,760	4067	18,220,160	3966	17,767,680
**Enco**	608	3,404,800	2014	11,278,400	2258	12,644,800	2023	11,328,800
**Bini**	594	1,330,560	1976	4,426,240	2192	4,910,080	2011	4,504,640
**Total**	29886	213,019,426	26757	177,792,518	28965	193,013,405	28845	191,694,08

Ipi = ipilimumab; Nivo = nivolumab; Pembro = pembrolizumab; Dab = dabrafenib; Tram = trametinib; Enco = encorafenib; Bini = binimetinib.

**Table 5:** Costs from adjuvant and metastatic treatment per financial year.

Year	Adjuvant		Palliative		Overall
	Cost (£)	Cost (%)	Cost (£)	Cost (%)	Cost (£)
*2019–2020*	93,786,434	31	213,019,426	69	306,805,859
*2020–2021*	80,662,992	31	177,792,518	69	258,455,510
*2021–2022*	89,522,536	32	193,013,405	68	282,535,941
*2022–2023*	99,210,086	34	191,694,081	66	290,904,167
*Total*	363,182,049	32	775,519,430	68	1,138,701,478
